# The Influence of Obesity, Ovariectomy, and Greenshell Mussel Supplementation on Bone Mineral Density in Rats

**DOI:** 10.1002/jbm4.10571

**Published:** 2021-11-14

**Authors:** Parkpoom Siriarchavatana, Marlena C Kruger, Matthew R Miller, Hong (Sabrina) Tian, Frances M Wolber

**Affiliations:** ^1^ School of Food and Advanced Technology Massey University Palmerston North New Zealand; ^2^ Department of Pharmacology, Faculty of Veterinary Science Chulalongkorn University Bangkok Thailand; ^3^ School of Health Sciences Massey University Palmerston North New Zealand; ^4^ Riddet Centre of Research Excellence Massey University Palmerston North New Zealand; ^5^ Cawthron Institute Nelson New Zealand; ^6^ Sanford Ltd. Auckland New Zealand; ^7^ Centre for Metabolic Health Research Massey University Palmerston North New Zealand

**Keywords:** BODY COMPOSITION, BONE MINERAL DENSITY, GREENSHELL MUSSEL, OBESITY

## Abstract

Obesity is considered to impair long‐term health by disturbing multiple physiological functions. However, it remains a controversial issue as to whether obesity has beneficial or detrimental effects on bone health in postmenopausal women. The aims of this study were to investigate the relationships between obesity and bone mineral density (BMD) under conditions of ovarian hormone deficiency in an animal model and to evaluate the potential health benefits of Greenshell mussel (GSM) on bone health. A total of 144 adult female Sprague–Dawley rats were fed from age 12 weeks on one of four diets (normal [ND]; ND + GSM; high fat/high sugar [HF/HS]; HF/HS + GSM; *n* = 36 per diet). At age 20 weeks, after a dual‐energy X‐ray absorptiometry (DXA) scan, 12 of the rats on each diet underwent ovariectomy (OVX) and the remaining rats were left intact. Twelve of the intact rats in each diet group were culled at age 26 weeks (short‐term cohort). The remaining rats were culled at age 48 weeks (long‐term cohort). Rats were DXA scanned before cull, then various fat pads were dissected. The results revealed that HF/HS rats and OVX rats dramatically increased body weight and fat deposition in correlation with leptin. In the long‐term cohort, vertebral spine BMD rapidly declined after OVX. At termination, the OVX rats had decreased plasma bone turnover markers of CTX‐1 and TRAP when compared with sham rats. Significantly higher BMD was found in OVX rats fed the HF/HS diet compared with ND, but this difference was not recapitulated in intact rats. BMD of right femur was significantly increased 5% to 10% by GSM in the short‐term cohort. The data demonstrated that obesity can be beneficial by increasing BMD in OVX rats, and this may extrapolate to postmenopausal women as adipocyte‐produced estrogen may slightly compensate for the reduction in ovarian hormones. Finally, the data showed that GSM may be beneficial to bone health by increasing BMD accrual. © 2021 The Authors. *JBMR Plus* published by Wiley Periodicals LLC on behalf of American Society for Bone and Mineral Research.

## 1 Introduction

The incidence of obesity has increased worldwide in the last five decades.^(^ ^1^ ^)^ It has been recognized as a predisposing cause for many chronic diseases, including hypercholesterolemia, hypertension, cardiac disease, and type 2 diabetes, giving it the status of a global public health burden.^(^ ^1^ ^)^ Osteoporosis is a disease of aging, and it shares a complex relationship with obesity. It used to be considered that obesity had a positive effect on osteoporosis based on a positive correlation between bone mineral density (BMD) and body mass index (BMI)^(^ ^2^, ^3^, ^4^ ^)^ and evidence from computed tomography showing that obese people have higher BMD in cortical and trabecular bone as well as increased trabecular bone at the distal radius and tibia.^(^ ^5^, ^6^ ^)^ Further support from a meta‐analysis study showed that low BMI correlated with hip fracture risk^(^ ^7^ ^)^ with obese adults having lower risk of hip fracture.^(^ ^8^ ^)^ Despite strong evidence of increased BMD in obese adults, the incidence of bone fracture is increased in obese children.^(9^ ^)^ Further, there is an increased fracture risk in obese people at the proximal humerus, upper legs, and ankles,^(^ ^10^, ^11^ ^)^ particularly in postmenopausal women.^(^ ^12^ ^)^ However, the fracture risk in these bone sites might be attributed to impairment of muscular function causing instability of movement in obesity.^(^ ^13^, ^14^ ^)^ Although obesity in older adults is accepted as being at least partly protective against osteoporosis, it is also clearly a risk factor for other chronic disorders, such as osteoarthritis, diabetes, cardiovascular disease, and metabolic syndrome. These contradictions in the benefits versus detriments of obesity have given rise to the term “obesity paradox,.”^(^ ^15^ ^)^

Homeostasis of bone is the equilibrium between bone formation supported by osteoblasts and bone remodeling driven by osteoclasts. From a perspective of cell origin, adipogenesis bias could threaten bone formation and result in bone loss because adipocytes and osteoblasts share a common precursor mesenchymal stem cell in bone marrow.^(^ ^16^ ^)^ This notion is evidenced by the fact that bone marrow of obese mice has more adipocyte infiltration.^(^ ^17^ ^)^ However, the increase of mechanical loading on bone in obesity can inhibit apoptosis and increase proliferation of osteoblasts and osteocytes^(^ ^18^, ^19^, ^20^ ^)^ by activation of the Wnt/β‐catenin signaling pathways.^(^ ^21^ ^)^ This could result in an increase in bone formation.

Another controversial factor is leptin, an adipokine secreted from adipocytes, which is significantly increased in the blood of obese people. On one hand, leptin has osteogenic effects by increasing osteoblastogenesis and suppressing adipocytic differentiation in human marrow stromal cells.^(^ ^22^ ^)^ Strong evidence supports the role of leptin in increasing BMD. Stephan and colleagues^(^ ^23^ ^)^ demonstrated that leptin administration resulted in an increase in femoral bone length, bone mineral content, and BMD in leptin‐deficient mice (ob/ob mice). Injection of leptin mitigated cancellous and trabecular bone loss in ovariectomized rats.^(^ ^24^ ^)^ However, other studies suggest that the effect of leptin on the central nervous system was different from its effect on peripheral tissues, resulting in the reduction of BMD.^(^ ^25^, ^26^ ^)^ Moreover, leptin indirectly activates osteoclastogenesis by activating macrophages to produce pro‐inflammatory cytokines.^(^ ^27^ ^)^ These cytokines in turn increase receptor activator of NF‐κB ligand (RANKL) expression in osteoblasts, which directly activates the process of osteoclast differentiation and subsequent bone resorption.^(^ ^28^ ^)^ Finally, adipocytes in large numbers, as occurs in obesity, can themselves increase the pro‐inflammatory cytokines TNF‐α, IL6, IL1β, IFN‐γ, MIP‐1, GRO‐α, and RANTES.^(^ ^29^ ^)^

The relationship between obesity and BMD is further complicated under postmenopausal circumstances. Estrogen deficiency is associated with not only fat deposition but also bone loss, leading to osteoporosis. In addition to the lack of estrogen causing calcium loss from bone, parathyroid levels are elevated correlating with body weight in obese menopausal women,^(^ ^30^, ^31^ ^)^ whereas circulating 25‐hydroxyvitamin D levels are low in obese people.^(^ ^32^, ^33^ ^)^ This evidence supports a correlation between bone loss and obesity in postmenopausal women. However, an increase in adiposity has a benefit on estrogen replacement because aromatase in adipocytes has the ability to produce estrogen as an alternative source for postmenopausal women.^(^ ^16^ ^)^ The controversy remains a highly debated subject and is unlikely to reach a consensus in clinical research. The current study addressed the issues described above by using an obese rat model compounded with the multiple challenges (high‐fat/high‐sugar diet, ovariectomy [OVX], metabolic dysregulation) and followed serial stages of the life cycles in order to further understand the changes in BMD in the context of obesity, postmenopause, and aging.

Supplements containing components of Greenshell mussel (GSM) have been widely reported to be a rich source of omega‐3 fatty acids^(^ ^34^, ^35^ ^)^ and to have beneficial anti‐inflammatory and anti‐arthritis effects.^(^ ^34^, ^36^, ^37^ ^)^ Omega‐3 fatty acids have been shown to protect against osteoporosis,^(^ ^38^ ^)^ although there is no published information to date regarding any potential benefits of GSM on bone health. The current study assessed whether GSM could benefit bone health by increasing BMD in growing rats or reducing bone loss in ovariectomized rats under either normal or obese conditions. The findings from this study combined with additional studies using a rat model as a tool for preclinical studies assessing the effect of GSM on obesity‐related disorders may clarify some of the ambiguities in the BMD‐obesity relationship and as well as in the obesity paradox.

## 2 Materials and Methods

### 2.1 Greenshell mussel and diet composition analyses

Flash‐dried powder from whole GSM meat was produced by Sanford Ltd (ENZAQ facility, Blenheim, New Zealand) using standard manufacturing processes and assessed for proximate composition in a commercial testing laboratory (Food Testing Laboratory of Cawthron Analytical Services, Nelson, New Zealand). To measure the stability of the diets, samples from each of the experimental diets used in the study were collected and frozen after mixing with mussel powder and the nutritive value analyzed according to the Association of Official Analytical Chemists (AOAC) methods for crude protein (AOAC 981.10), total fat (AOAC 948.15), moisture at 105°C (AOAC 950.46), and ash (AOAC 920.153). Carbohydrate content was determined by calculation (100% − % crude protein – % total fat – % moisture – % ash). An aliquot of the total lipid extract from the GSM powder was transmethylated in methanol/chloroform/hydrochloric acid (10:1:1, v/v/v) for 1 hour at 100°C. After the addition of water, the mixture was extracted three times with hexane/chloroform (4:1, v/v) to obtain fatty acid methyl esters (FAME). Samples were made up to 1 mL with an internal injection standard (19:0 FAME) and analyzed by gas chromatography mass spectrometry (GC‐MS) according to AOAC 963.22.

### 2.2 Animals and study design

A total of 144 female Sprague–Dawley rats aged 12 weeks were obtained from the Small Animal Production Unit at Massey University (Palmerston North, New Zealand), randomized into groups, and fed test diets for 14 weeks (short‐term cohort, 48 rats) or 36 weeks (long‐term cohort, 96 rats) after a 1‐week acclimatization period. Animals were maintained in conventional systems and singly housed in plastic “shoebox” cages with high‐top wire lids, using heat‐treated aspen wood shavings as bedding. The rat rooms were kept at 22 ± 1°C, 45% to 55% humidity, and a 12‐hour light/dark cycle. The numbers of rats per test group were selected calculating a power (1‐β) of 0.8 and a type 1 error rate (α) of 5% for the key parameter of BMD using data from our research group's previous rat studies. All aspects of the study were approved by Massey University Animal Ethic Committee (MUAEC protocol/approval 16/112).

Normal versus high‐fat/high‐sugar diets were used to allow comparisons between normal‐weight and obese rats groups that these diets generated. Each base diet type (Specialty Feeds, Glen Forrest, Western Australia) was obtained as a single batch, packaged in vacuum‐sealed plastic pouches, and used within 9 months of receipt. Diets were stored frozen until needed. All diets were designed to contain 15% protein as is standard for AIN‐93M. The proportion of GSM was selected on the assumption that, extrapolated to the human diet, one meal out of each three meals per day would source the protein from GSM; therefore, for the rat diets, one‐third of the protein was sourced from GSM for the GSM‐containing diets. After thawing a pouch in the refrigerator, diets designed to incorporate GSM had GSM powder added in a ratio of one part GSM/nine parts base diet. Diets were then stored refrigerated during the week of use. Subsamples of each diet were taken periodically for further chemical analysis.

Each day, uneaten diet from each individual feeder was weighed and discarded, and feeders were cleaned and filled with new food, which was weighed and recorded to measure food intake. The test diets, designed to meet or exceed all AIN‐93M requirements, were:

- normal control diet (ND) containing 5% total fat (from soy oil), 5% sucrose, and 15% total protein (from casein);
- normal control diet supplemented with GSM (ND + GSM) containing 5% total fat (84% from soy oil/16% from GSM), 5% sucrose, and 15% total protein (66% from casein/33% from GSM);
- high‐fat/high‐sugar diet (HFHS) containing 30% total fat (50% from soy oil/50% from lard), 30% sucrose, and 15% total protein (from casein);
- high‐fat/high‐sugar diet supplemented with GSM (HFHS + GSM) containing 30% total fat (49% from soy oil/49% from lard/1% from GSM), 30% sucrose, and 15% total protein (66% from casein/33% from GSM).

### 2.3 Dual‐energy X‐ray absorptiometry (DXA) scans

DXA whole‐body and regional scans to generate BMD and fat mass/lean mass data in the short‐term cohort were performed at baseline (age 12 weeks) and after 8 and 14 weeks on the experimental diets (age 20 and 26 weeks). The long‐term cohort schedule was similar to the short‐term cohort but with an additional scan after 36 weeks on the experimental diets (age 48 weeks). For both cohorts, after the baseline DXA scan, rats were randomized into diet groups, ensuring that there were no significant differences between groups in basal body weight and no significant differences in lumbar spine and whole‐body bone mineral densities. Rats in the long‐term cohort were further randomized into surgical groups within their diet groups, with randomization again based on eliminating significant differences in body weight and BMD.

Anesthesia and DXA scans were carried out as described previously.^(^ ^39^ ^)^ BMD was evaluated with a Hologic Discovery, a bone densitometer (Hologic Inc., Bedford, MA, USA). Quality‐control checks were performed before scanning the animals, and the acceptance of coefficient of variance was 0.98 to 1.01. Rats were positioned supine with right angles between the spine and femurs. All scans used a high‐resolution mode.

### 2.4 Surgical procedures and euthanasia

In the long‐term cohort, at the age of 20 weeks, half of each group (12 of 24 rats) underwent ovariectomy and the other half a sham surgery as described previously.^(^ ^39^ ^)^At the end of each cohort, all rats were subjected to euthanasia by undergoing deep anesthesia followed by exsanguination. Samples including different fat pads (inguinal fat, interscapular brown fat, retroperitoneal fat and perigonadal fat) were dissected and weighed.

### 2.5 Plasma analysis for leptin and bone biomarkers

At the end of the study, anesthetized rats underwent cardiac puncture. Blood was drawn and collected in EDTA‐anticoagulant tubes. Plasma was separated by centrifugation at 2500 rpm (Herareus Megafuge 1.0R, Thermo Fisher Scientific, Waltham, MA, USA) and kept at −80°C until analysis. All analytes were measured using ELISA kits except for tartrate‐resistant acid phosphatase (TRAP), for which individual chemical reagents for a chromogenic assay performed as published elsewhere^(^ ^40^ ^)^ were purchased from Sigma‐Aldrich (St. Louis, MO, USA). Leptin was measured using Quantikine Kits (R&D Systems, Minneapolis, MN, USA). CTX‐I assay kits were obtained from Cloud‐Clone Corp. (Houston, TX, USA). Samples were performed in duplicate, and reactions were measured using a microplate reader (Multiskan FC, Thermo Fisher Scientific, Vantaa, Finland)

### 2.6 Data analysis

Body weight, feed intake, and fat pads were analyzed by one‐way ANOVA followed by the least significant difference test. Student's *t* test was used to identify statistically significant differences in fat pad weights between the short‐term rats and the sham rats in the long‐term cohort or the sham rats and OVX rats. Data from serial DXA scans were analyzed by repeated measure method composed of three effects: diet (ND/HFHS), GSM (yes/no), and surgical procedure (OVX, sham). Pearson correlation was used to evaluate relationships between body composition and BMD. Bone markers in blood collected from the rats at termination were analyzed by two‐way ANOVA. Statistical analysis was performed using IBM (Armonk, NY, USA) statistic software version 25, and differences were considered significant at *p* < 0.05.

## 3 Results

### 3.1 Analysis of diet composition

Protein was equivalent in all diets (Table 1), {TBL 1} but the partial substitution of carbohydrate with fat in the HFHS and HFHS + GSM diets increased the energy levels to 24.9 and 22.9 kj/g compared with the ND and ND + GSM diets (16.9 and 16.8 kj/g, respectively). Added fat in the HFHS diet increased saturated fatty acid (SFA) and monounsaturated fatty acid (MUFA) but not polyunsaturated fatty acid (PUFA). Two important omega‐3 fatty acids particularly associated with bone‐health protection, docosahexaenoic acid (DHA) and eicosapentaenoic acid (EPA), were increased in diets containing GSM, but there was no change in the omega‐6 fatty acids (linoleic acid and arachidonic acid) (Table 1). Ash content of GSM powder contains high amounts of inorganic substances, including sodium and calcium. Inclusion of GSM powder to the base diets resulted in an increase of 2% to 4% ash in those diets.

**Table 1:** Nutritive Value of the Four Different Diets in the Study

Diet composition	ND	ND + GSM	HFHS	HFHS + GSM
Proximate composition (g/100 g)				
Fat	5.4	5.1	31.3	30.1
Ash	2.9	4.7	2.7	4.1
Moisture	8.3	8.2	3.7	3.3
Crude protein	13.5	13.3	13.5	12.6
Carbohydrate	69.9	68.7	48.8	49.9
Fatty acid profile (% fatty acids)				
C16:0 palmitic acid	11.2	12.1	18.2	17.8
C18:0 stearic acid	4.8	4.7	10.4	10
C18:1n7 vaccenic acid	1.5	1.7	1.9	1.9
C18:1n9c oleic acid	21.9	20.6	28.2	27.8
C18:2n6c linoleic acid	48.6	45.5	31.7	31.6
C18:3n3 alpha linolenic acid (ALA)	6.2	5.8	4.1	4.2
C20:4n6 arachidonic acid (AA)	<0.1	<0.1	<0.1	<0.1
C20:5n3 eicosapentaenoic acid (EPA)	<0.1	1.2	<0.1	0.31
C22:6n3 docosahexaenoic acid (DHA)	<0.1	0.93	<0.1	0.21
∑SFA	16	16.8	28.6	27.8
∑MUFA	23.4	22.3	30.1	29.7
∑PUFA	54.8	53.43	35.8	36.32
∑n‐3 PUFA	6.2	7.93	4.1	4.72
∑n‐6 PUFA	48.6	45.5	31.7	31.6

1 Diet samples were taken during the experimental period and sent to Cawthron Institute at the end of the study. The analysis was performed according to the Association of Official Analytical Chemists (AOAC) methods.

### 3.2 Body weight gain and food consumption

**Fig 1:**
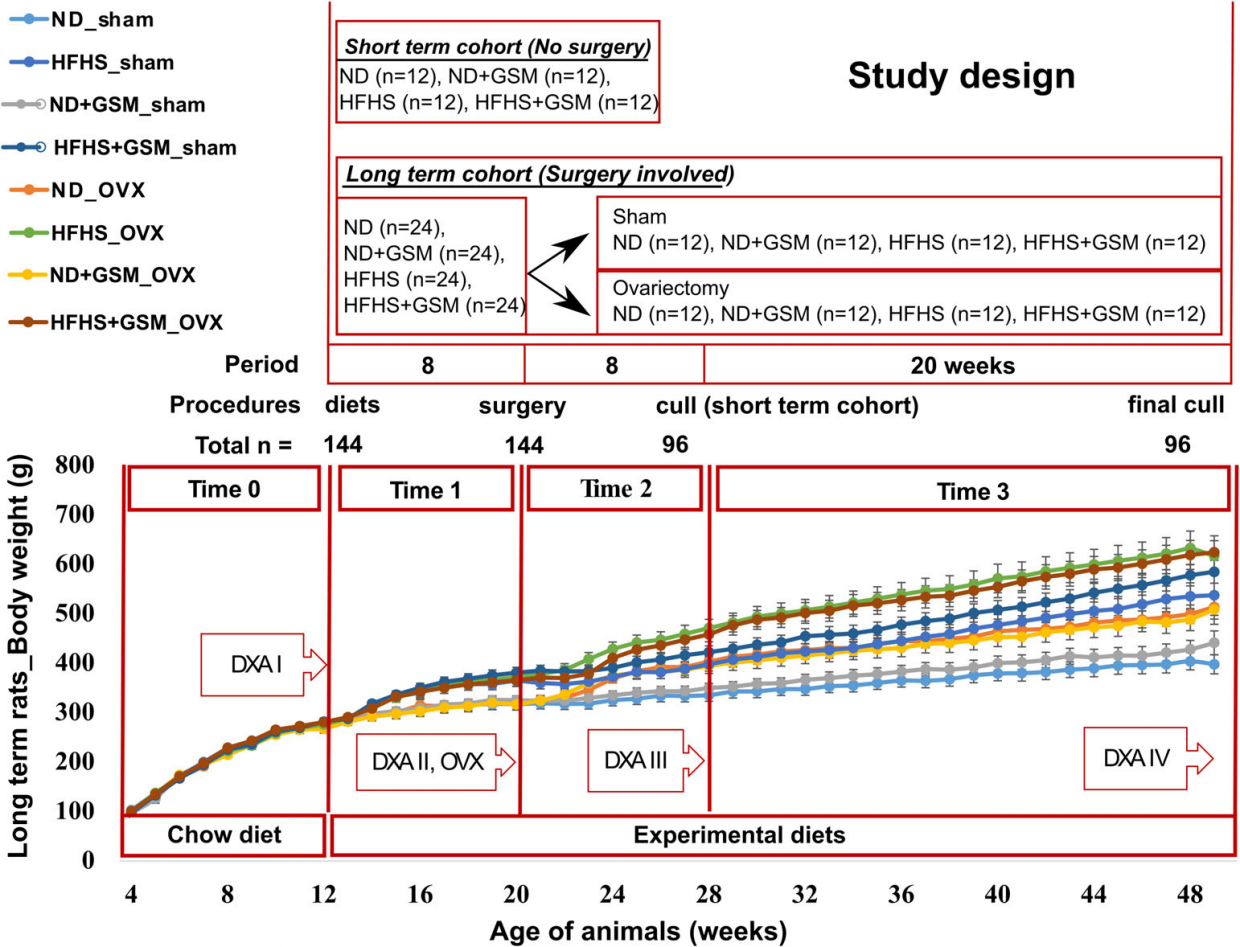
Study plan and body weight (BW) gain in rats along the long‐term cohort. Individual rat body weights were recorded in grams every week. Group mean ± SE are shown, plotted in the line graph. ND = normal control diet; HFHS = high‐fat/high‐sugar diet; OVX = ovariectomy; DXA = dual energy X‐ray absorptiometry scanning. The periods of the study were specified as “Time”; Time 0 = rat growing period on standard chow; Time 1 = experimental diets provided for 8 weeks; Time 2 = 8 weeks after the surgical procedure; Time 3 = termination of the study.

Time 0 (T0) designates the period of growing rats on standard chow from age 4 to 12 weeks, during which the rate of body weight (BW) gain of almost 3 g/d was similar across all groups in the long‐term cohort (Fig. 1 {FIG1}and Table 2). {TBL 2} The experimental diets were introduced to the animals at week 12 (the end of T0). At this stage, the range of mean BW across all groups was 267.37 to 280.84 g with no significant difference between groups (*p* = 0.911). The rate of mean BW gain in the following periods (T1 to T3) declined consecutively, especially in intact rats on the normal control diet (ND), whereas BW gain in the OVX rats increased immediately after the surgical procedure (T2) before returning to the rate of gain observed in T1.

**Table 2:** Changes in Food Consumption and Body Weight

Parameters	Sham				OVX				*p V*alues
	ND	ND + GSM	HFHS	HFHS + GSM	ND	ND + GSM	HFHS	HFHS + GSM	
BW 12th week	273.73 ± 8.53	276.06 ± 6.20	280.84 ± 7.01	281.62 ± 7.54	275.64 ± 8.20	267.37 ± 8.96	276.51 ± 7.93	280.70 ± 6.41	NS
BW 20th week	317.30 ± 10.82^ab^	325.39 ± 8.69^abcd^	365.21 ± 12.28^bcde^	380.91 ± 10.97^e^	320.58 ± 7.72^abc^	316.68 ± 11.37^a^	372.09 ± 13.61^de^	365.50 ± 11.78^cde^	<0.001
BW 28th week	335.54 ± 12.40^a^	349.75 ± 9.63^ab^	397.77 ± 13.73^bc^	422.59 ± 12.54^cde^	403.13 ± 11.05^bcd^	394.58 ± 15.24^abc^	470.91 ± 17.97^e^	458.34 ± 14.92^de^	<0.001
BW the end	403.89 ± 17.30^a^	428.18 ± 18.05^a^	535.38 ± 27.16^bc^	578.15 ± 21.78^bc^	499.20 ± 18.56^ab^	488.27 ± 22.36^ab^	633.35 ± 34.01^c^	619.05 ± 29.34^c^	<0.001
Weight gain (g/d)									
Time 0	2.71 ± 0.11	2.85 ± 0.10	2.91 ± 0.09	2.93 ± 0.09	2.82 ± 0.09	2.65 ± 0.14	2.80 ± 0.10	2.85 ± 0.11	NS
Time 1	0.65 ± 0.08^a^	0.67 ± 0.08^a^	0.99 ± 0.10^ab^	1.37 ± 0.14^b^	0.66 ± 0.07^a^	0.70 ± 0.15^a^	1.26 ± 0.20^ab^	1.26 ± 0.27^ab^	<0.001
Time 2	0.54 ± 0.05^a^	0.56 ± 0.06^a^	0.70 ± 0.09^a^	0.69 ± 0.09^a^	1.78 ± 0.10^bc^	1.32 ± 0.10^b^	1.74 ± 0.15^bc^	1.89 ± 0.17^c^	<0.001
Time 3	0.48 ± 0.05^a^	0.55 ± 0.06^ab^	0.87 ± 0.10^bcd^	0.94 ± 0.09^cd^	0.68 ± 0.07^abc^	0.64 ± 0.05^abc^	1.10 ± 0.14^d^	1.06 ± 0.11^d^	<0.001
Feed intake (g/d)									
Time 1	16.72 ± 0.53^c^	15.90 ± 0.56^bc^	12.71 ± 0.47^a^	13.76 ± 0.37^ab^	16.97 ± 0.51^c^	15.55 ± 0.68^bc^	13.28 ± 0.54^a^	12.57 ± 0.41^a^	<0.001
Time 2	16.17 ± 0.44^b^	15.79 ± 0.56^b^	11.94 ± 0.42^a^	12.13 ± 0.38^a^	19.53 ± 0.48^c^	18.61 ± 0.62^c^	14.76 ± 0.47^b^	14.36 ± 0.48^b^	<0.001
Time 3	16.19 ± 0.48^b^	16.22 ± 0.53^b^	12.86 ± 0.46^a^	13.43 ± 0.43^a^	16.81 ± 0.45^b^	16.48 ± 0.37^b^	13.61 ± 0.58^a^	13.68 ± 0.49^a^	<0.001
Energy intake (Kcal/d)									
Time 1	59.93 ± 1.91^ab^	57.00 ± 2.00^a^	63.81 ± 2.38^ab^	69.08 ± 1.84^b^	60.82 ± 1.83^ab^	55.75 ± 2.45^a^	66.64 ± 2.71^b^	63.08 ± 2.04^ab^	<0.001
Time 2	57.96 ± 1.58^ab^	56.60 ± 2.01^a^	59.94 ± 2.11^ab^	60.89 ± 1.90^ab^	70.02 ± 1.71^c^	66.71 ± 2.21^bc^	74.07 ± 2.34^c^	72.08 ± 2.39^c^	<0.001
Time 3	58.03 ± 1.72^a^	58.16 ± 1.88^a^	64.54 ± 2.30^abc^	67.39 ± 2.15^bc^	60.27 ± 1.61^abc^	59.09 ± 1.34^ab^	68.31 ± 2.90^c^	68.65 ± 2.44^c^	<0.001

2 The food consumption and body weight of different time points from the intact rats (sham) and the ovariectomized rats (OVX) in different diet groups are summarized. Values are mean ± SE. The significant differences across all conditions (diets and surgical procedures) were determined by one‐way ANOVA and *p* values are indicated in the last column. In case no statistical significance (NS) was found, then no multiple comparison was performed. Significance is recognized when *p* < 0.05, and superscript letters indicate the least significant difference test. Values that do not share the same superscript letters are significantly different.

During the first 8 weeks on the experimental diets before surgery (T1), rats fed HFHS consumed 20% less food by weight than those fed ND but had a 12% higher kilojoule intake due to the HFHS diets being more energy‐dense (Table 2). As a result, the HFHS rats had an 82% higher rate of mean daily BW gain compared with ND rats. Inclusion of GSM during this period slightly increased weight gain, but this was not significant. After sham operation or OVX at the end of T1, during the next 8‐week period (T2) feed intake, energy intake and BW gain were significantly higher in OVX rats compared with sham rats (BW gain in sham = 0.54 to 0.70 g/d versus OVX 1.32 to 1.89 g/d, *p* < 0.001). For the last period of the study (T3), voluntary food consumption rate by OVX rats reduced to a similar rate as sham rats, but their BW gain continued to be higher than sham rats and subsequently OVX rats had higher BW at the end of the study, especially those fed HFHS. The OVX procedure increased total final BW by 24% and 18%, respectively, in rats fed ND and HFHS compared with their sham counterparts (*p* < 0.001), but only 14% and 7%, respectively, in rats fed ND + GSM and HFHS+GSM, suggesting that regardless of diet type, the inclusion of GSM partially mitigated the weight gain caused by OVX, although this did not reach statistical significance.

### 3.3 Changes in body composition

**Fig 2:**
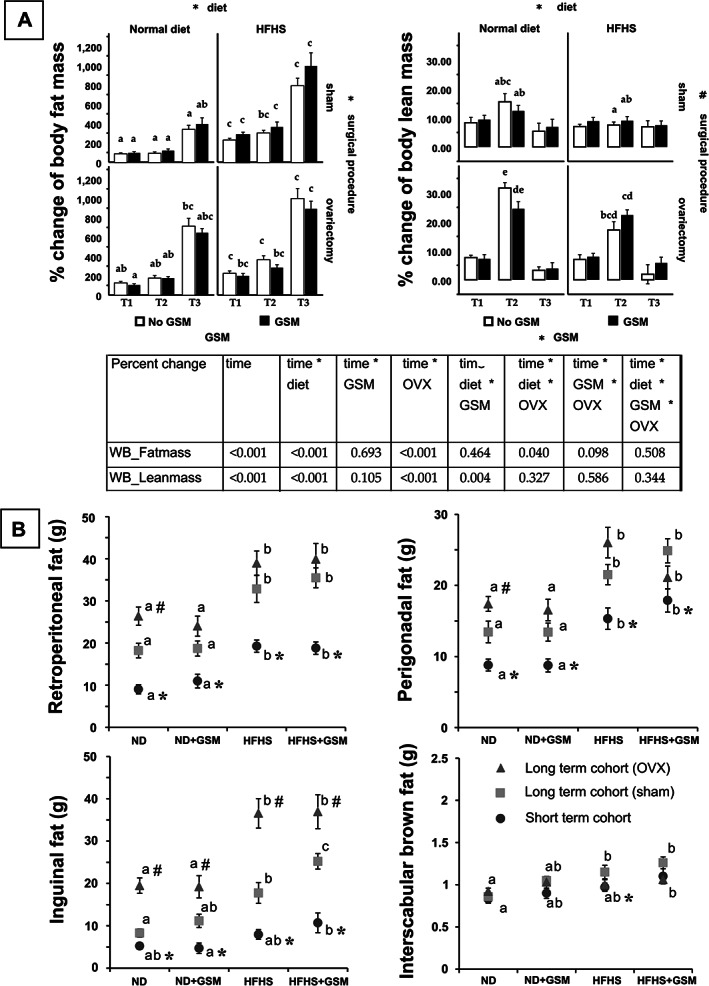
Changes in body composition. Body composition was measured by dual‐energy X‐ray absorptiometry in the long‐term cohort rats (12 to 48 weeks old) (*A*). Then data were statistically analyzed by repeated measured method and a summary of *p* value was reported in the table. The signs (* or #) adjacent to the name of the effects (diet, surgical procedure, Greenshell mussel [GSM]) indicate significant differences. In case of an interaction effect, the same sign is present on those effects. One‐way ANOVA and a multiple comparison (least significant difference test) were also used to analyze the data across all groups. The differences of letters indicate significant differences. At the end of each study, all rats were euthanized and each type of fat pad was harvested and weighed (*B*). Data are summarized in box‐whisker graphs that represent mean ± SE. One‐way ANOVA and the least significant difference test were used to analyze data across the groups, then different superscript letters were given to indicate statistical significance. Independent *t* test was used to compare the data in the same diet condition. An asterisk (*) shows significant difference when comparing the short‐term cohort with the long‐term (sham). Hashtag (#) was used instead for comparison between the sham rats and OVX rats. All statistical significance was determined by *p* value < 0.05. ND = normal control diet; HFHS = high‐fat/high‐sugar diet; OVX = ovariectomy. Short‐term cohort = non‐operated rats euthanized at 26 weeks of age; long‐term cohort (sham) = intact rats euthanized at 48 weeks of age; long‐term cohort (OVX) = ovariectomized rats euthanized at 48 weeks of age.

In the long‐term cohort (Fig. 2*A*), {FIG2} body fat mass of all rats increased significantly over time. Both HFHS and OVX independently caused significant increases in the acquisition of body fat mass. During T1 (ages 12 to 20 weeks), body fat mass approximately doubled in ND groups and tripled in HFHS groups. This increase was recapitulated during T2 after surgery (ages 20 to 28 weeks), and was even more pronounced during T3 (ages 28 to 48 weeks), when body fat mass increased nearly 10‐fold; the effect was greatest in OVX rats fed HFHS. Interestingly, OVX caused a significant increase in body lean mass over 8 weeks (T2), but no further gains in lean mass occurred during the 20‐week T3 period. There was a significant interaction effect of diet and GSM on body lean mass. At T2, HFHS reduced the increase in lean mass acquired by ND rats. Adding GSM in ND caused a reduction in lean mass, whereas being combined with HFHS resulted in a slight increase in lean mass. However, the latter two findings were trends and did not reach statistical significance.

At the end of the short‐term and long‐term studies, all rats were subjected to necropsy and both visceral and subcutaneous fat pads were harvested and weighed (Fig. 2*B*). Data from the short‐term cohort who did not undergo sham or OVX surgery were compared with the sham rats of the long‐term cohort, whereas the OVX rats were compared against the sham rats in the same cohort. As expected, feeding HFHS diets to rats for 14 or 36 weeks resulted in a significant increase in retroperitoneal (32.84 ± 3.17) and perigonadal (21.52 ± 1.43) as well as inguinal (subcutaneous) white fat pad weights (17.76 ± 2.48) compared with ND but did not consistently alter the much smaller pad of interscapular brown fat (1.15 ± 0.08); the weight of the brown fat differed significantly only between the ND and HFHS + GSM groups in the short‐term cohort and the long‐term sham cohorts. Likewise, age significantly increased the weight of the white but not brown fat pads. OVX significantly increased the abdominal fat pads only in rats fed ND, but significantly increased the subcutaneous fat pad weight in all four diet groups. (Fig. 2*B*).

### 3.4 Changes in bone mineral density

**Fig 3:**
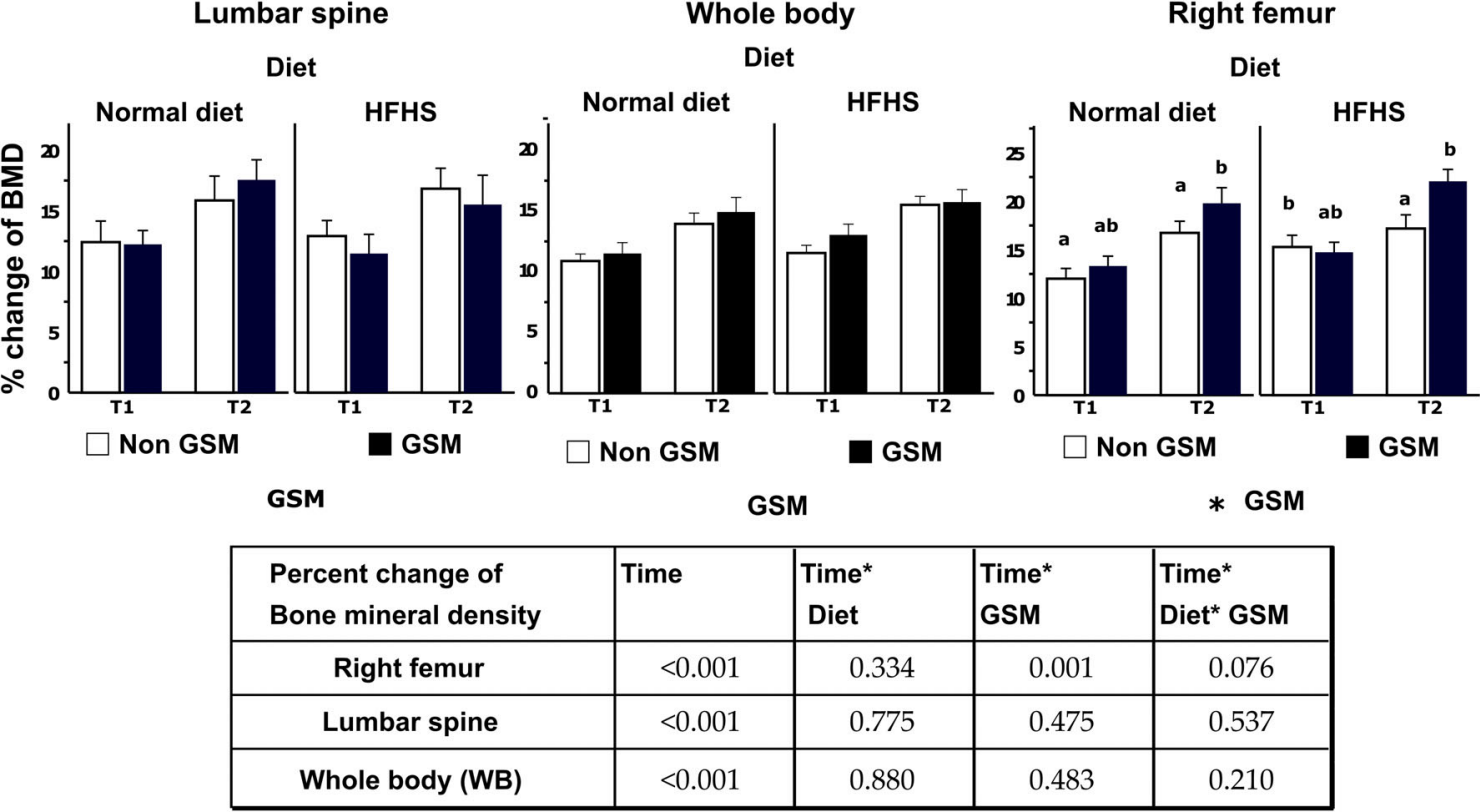
Percent change of bone mineral density in short‐term cohort rats (12 to 26 weeks old). The individual chart represents the different bone sites, which are lumbar spine (L_1_ to L_4_), whole body (WB), and right femur. Bars and error bars represent mean and SE (*n* = 12) of percent change between the baseline of bone mineral density (BMD) at week 12 and the particular time point (T1 or T2, which is week 20 and week 26, respectively). Repeated measure analysis was used to analyze statistically significant differences between the time points and those two effects (diet and GSM). An asterisk is indicated on graphs next to the name of the effect when the effect is significant. Letters (a or b or ab) are provided as possible if there is a statistical difference when comparing the mean of the different groups of rats in the same period by one‐way ANOVA, and least significant difference method was used for multiple comparison test. A table summary from repeated measured analysis shows *p* values that are significant at <0.05. ND = normal control diet; HFHS = high‐fat/high‐sugar diet; OVX = ovariectomy; GSM = Greenshell mussel.

Bone mineral density was measured in three different scans: whole body, lumbar spine, and right femurs. In the short‐term cohort (Fig. 3), {FIG3} whole‐body BMD increased with time between baseline (age 12 weeks) to age 20 weeks (T1) and 26 weeks (T2) regardless of diet or GSM. Changes in BMD of spine and femurs were proportionally greater than whole‐body BMD. Of interest was the finding that inclusion of GSM in the HFHS diet significantly increased femur BMD during the final 6 weeks of the study (T2).

**Fig 4:**
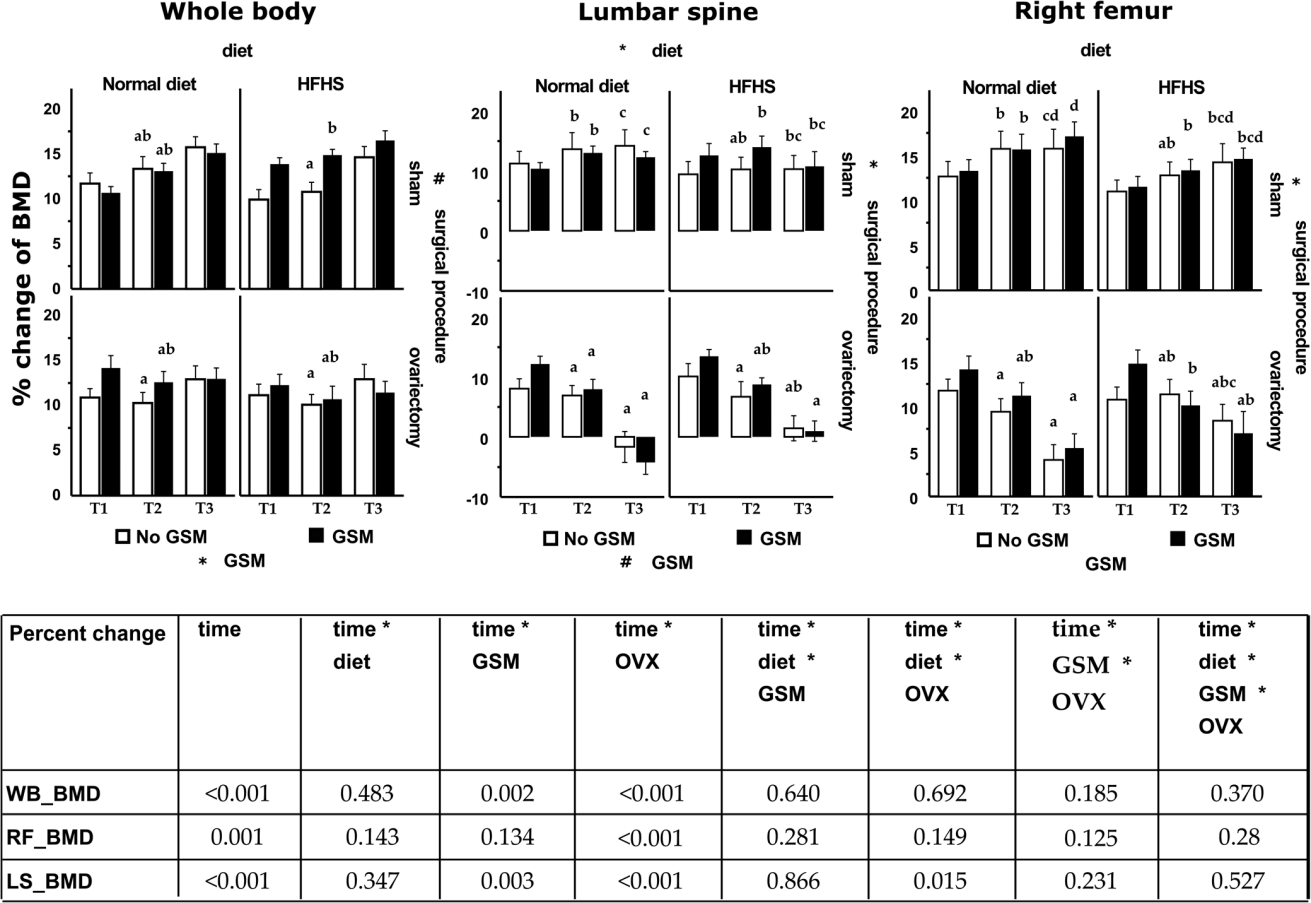
Percent change of bone mineral density in long‐term cohort rats (12 to 48 weeks old). The individual chart represents bone mineral density (BMD) of the different bone sites, which are whole body (WB), lumbar spine (L_1_ to L_4_), and right femur. Bars and error bars represent means and SE (*n* = 12) of percent change between the baseline of bone mineral density (BMD) at week 12 and the particular time point (T1 or T2 or T3, which is week 20, 28, and 48, respectively). Repeated measure analysis was used to analyze statistically significant differences between the time points and those three effects (diet, GSM, and OVX). A sign (* or #) was labeled close to the name of the effects when statistical significance of the main effect was detected. The similar sign shown on different effects means the significance of the interaction effect between those. One‐way ANOVA and least significant difference methods were used to analyze data between groups within one particular time point, then differences of superscript letters show statistical significance. A table summary from repeated measures analysis shows *p* values, which is significant <0.05. ND = normal control diet; HFHS = high‐fat/high‐sugar diet; OVX = ovariectomy; GSM = Greenshell mussel.

In the long‐term cohort, OVX was the main determinant causing BMD to fail to increase in tandem with body mass; in particular, only OVX rats lost BMD in the lumbar spine and the right femur (Fig. 4). {FIG4} GSM showed a trend to increase whole‐body, lumbar spine, and right femur BMD at T1 in all groups but ND sham rats, and a significant difference was also detected in sham rats on HFHS at T2 of the whole‐body scan. There was an interaction effect between diet and surgical procedure in the lumbar spine BMD changes during T3. All sites of BMD in sham rats fed ND tended to be higher compared with sham HFHS rats, but the opposite effect was observed in OVX rats. Likewise, in the right femurs, only OVX had a significant effect, resulting in a significant reduction in BMD during T3.

### 3.5 Relationships between obesity and bone mineral density

**Fig 5:**
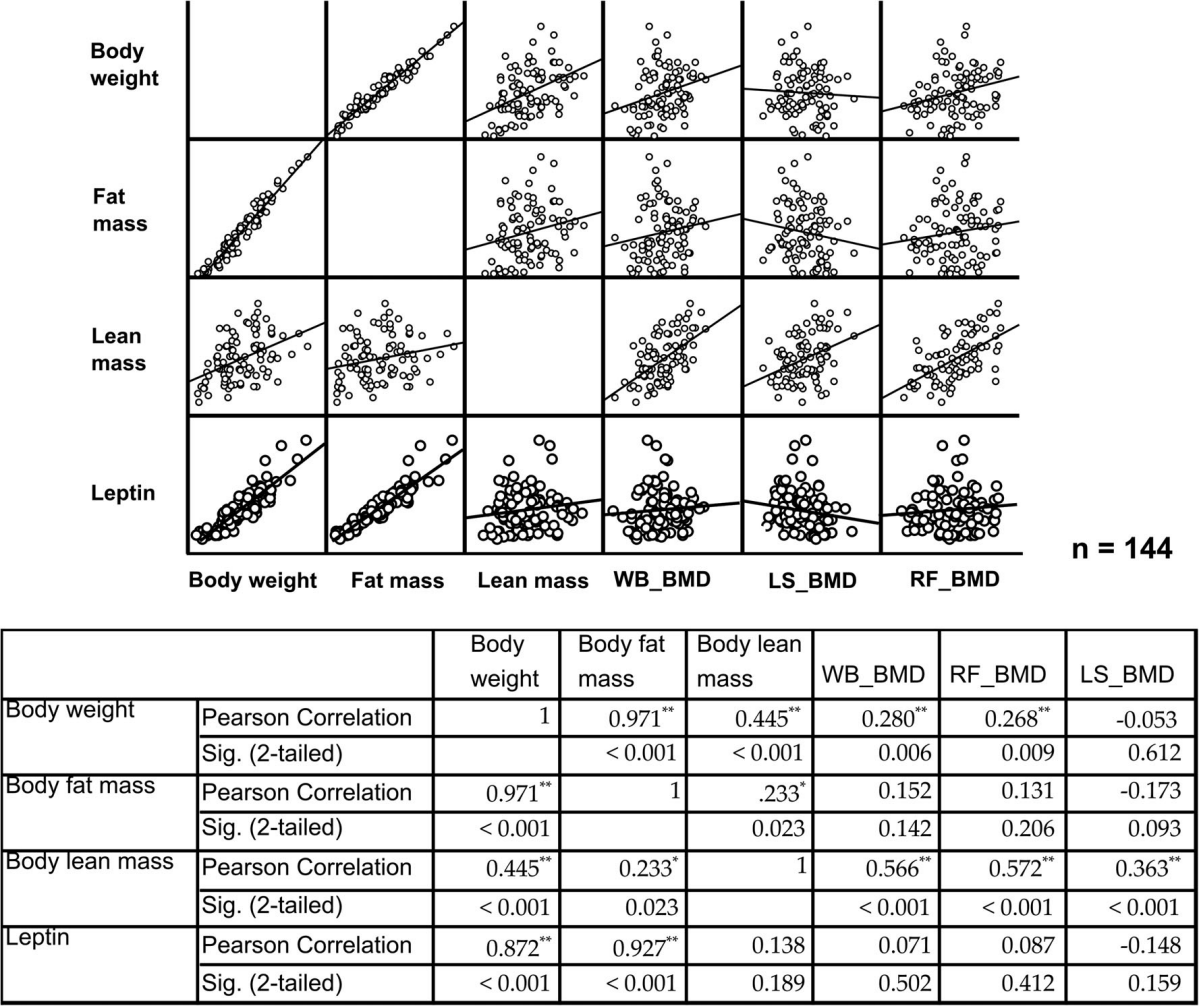
Correlation of body weight or fat mass or lean mass or leptin against bone mineral density of different bone sites. The data at the final time point (T3) from all rats of the long‐term cohort was pooled together and analyzed. Correlation is presented in both figures and values from Pearson correlation method. Statistical significance was set at *p* < 0.05 and asterisk (*) was indicated. **p < 0.01; ***0.001. WB = whole body; LS = lumbar spine; RF = right femur.

**Fig 6:**
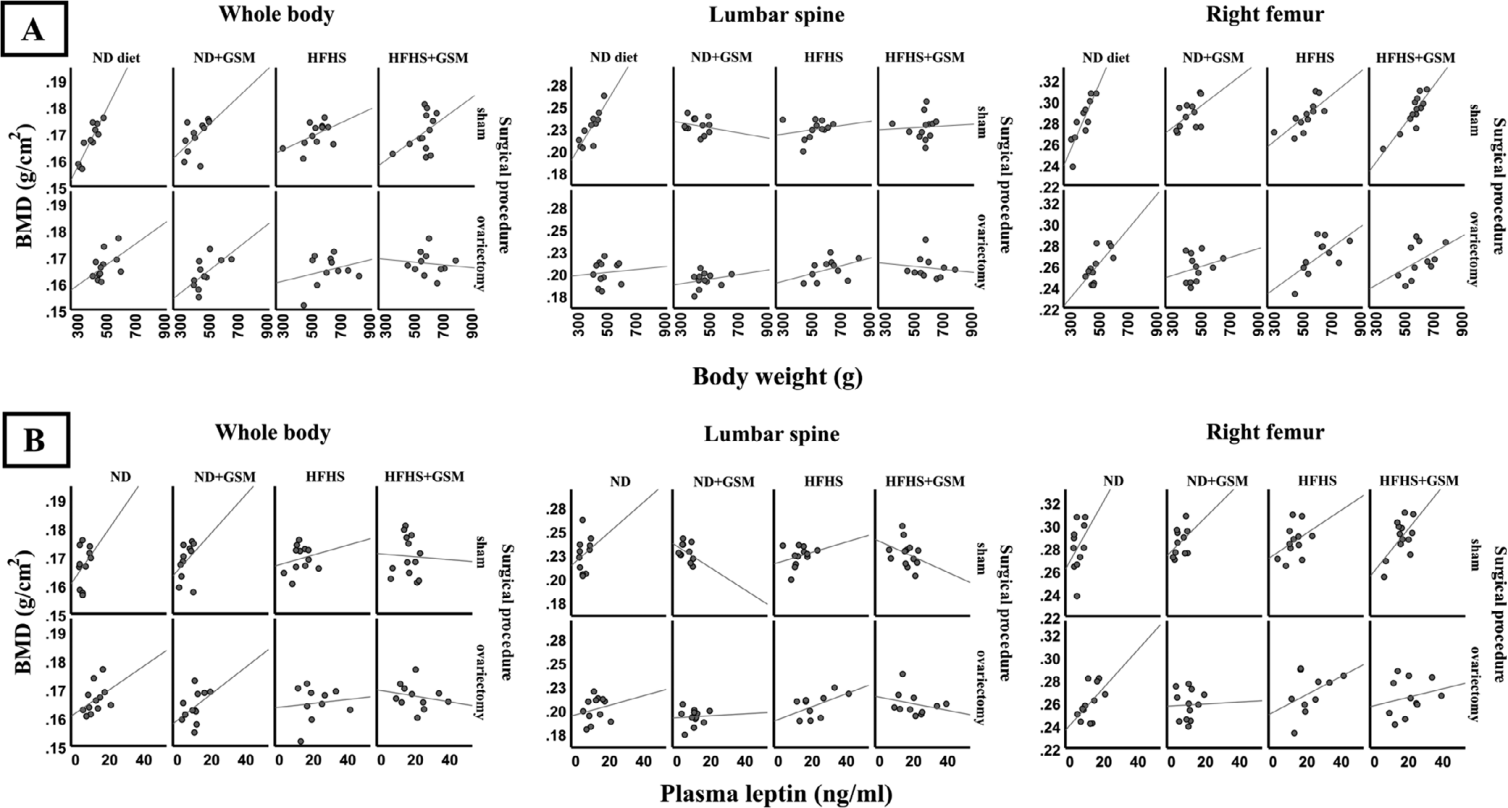
Correlation of body weight or leptin with bone mineral density in the individual group. Data at the final time point (T3) of the long‐term cohort was stratified in the different condition groups and correlation analysis was performed. Correlation between body weight and bone mineral density (BMD) or between leptin and BMD is present in (*A*) and (*B*), respectively. The correlation table is provided in the Supplemental Material S1.

To understand the relationship between obesity and BMD, data from T3 were interrogated and the correlations between BMD of individual bone sites and body weight, fat mass, lean mass, or leptin were graphed (Fig. 5). {FIG5} BW had minimal correlation with whole‐body and right femur BMD at 0.280 and 0.268, respectively. Extracting BW into fat mass and lean mass resulted in a higher correlation of body lean mass with BMD in all scans, whereas body fat mass correlated poorly with BMD. Leptin, an adipokine produced by adipocytes, was highly correlated with fat mass (0.927, *p* < 0.001) but showed no correlation with any BMD scans. Then BMD data were segregated into each group and correlation curves of BMD against BW or plasma leptin were depicted (Fig. 6 {FIG6}and Supplemental Fig. S6). As can be found in the graphs, there were tightly positive correlations between BMD and BW in only the sham rats on ND. Introducing other interventions of either GSM, HFHS, or OVX caused some modification of this pattern and possibly explained the interference of their effect on BMD. In the whole‐body BMD, the positive correlation as shown by trend line turned downward in OVX rats on HFHS and similar downturns were also found in most groups of lumbar spine BMD.

### 3.6 Changes in plasma bone biomarkers

**Fig 7:**
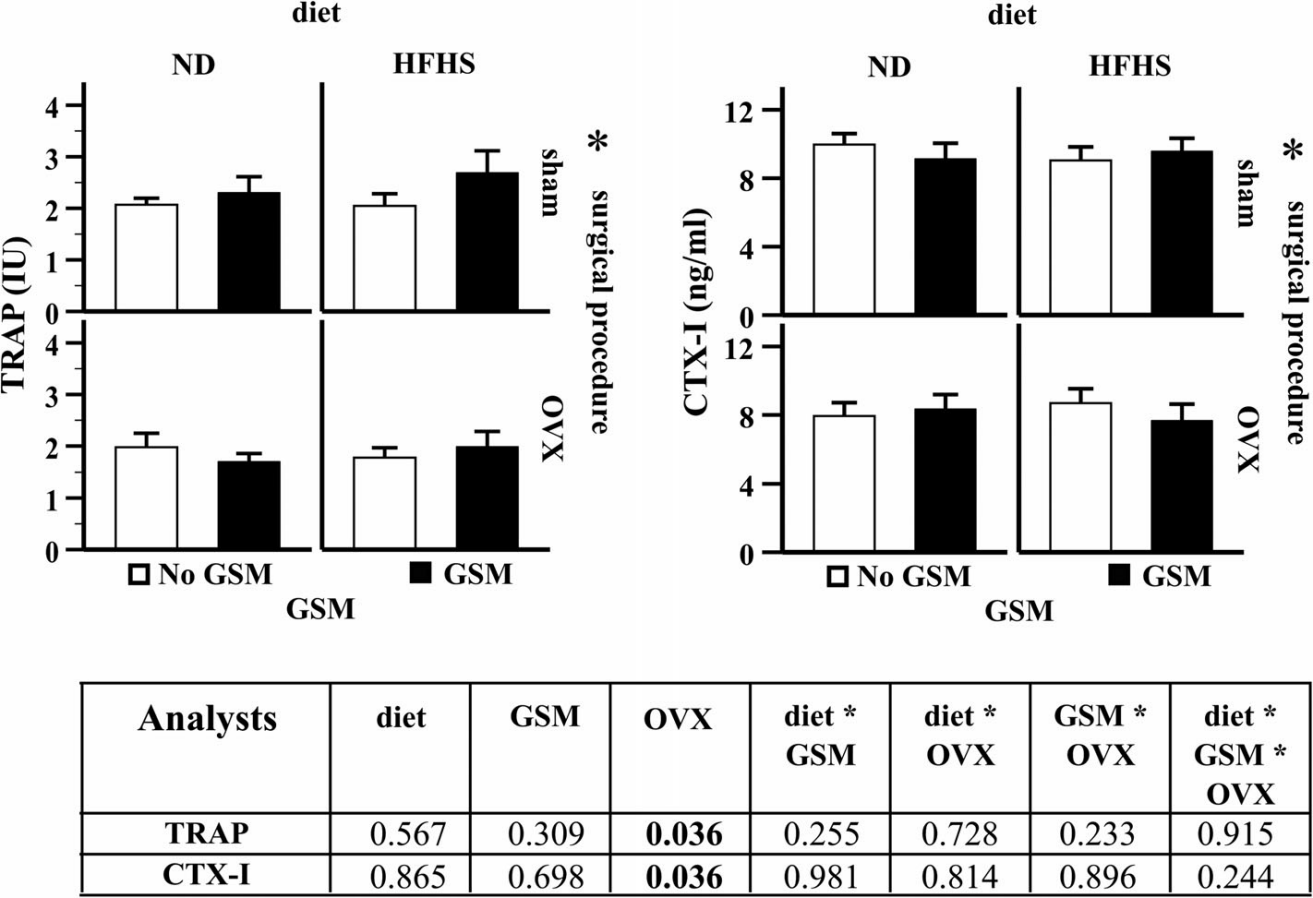
Changes in plasma bone markers. Plasma analysis was measured in the rats at 48 weeks of age. Bar graphs with error bars show means ± SE. Two‐way ANOVA was used to evaluate the influence of effects of diet, surgical procedure, and GSM. If the effect was statistically significant, and asterisk (*) is indicated. A table summary from two‐way ANOVA analysis shows *p* values that are significant at <0.05. ND = normal control diet; HFHS = high‐fat/high‐sugar diet; OVX = ovariectomy; GSM = Greenshell mussel.

Bone metabolism was systematically altered by ovariectomy as can be found in the reduction of plasma TRAP (*p* = 0.036) and CTX‐I (*p* = 0.036) (Fig. 7). {FIG7} All sham rats expressed >2 units of TRAP, whereas all OVX rats expressed <2 units. Similarly, CTX‐I levels in the sham rats expressed >9 ng/mL, whereas the OVX rats produced <9 ng/mL.

## 4 Discussion

Our study showed that GSM supplementation increased BMD of the right femur, especially in rats in the short‐term cohort. The statistically significant difference of this data is consistent even after adjusting BMD with BW. However, the effect was very mild (only 5% differences when compared with non‐GSM diet), inconsistent with comparative BMD in other body sites, and transient as this effect disappeared at the same time (T2) and the later time (T3) in the long‐term cohort. It is possible that with this very small effect size, a larger sample size would be needed to reveal a consistent result. On the other hand, it might be irrelevant to expect more than a minor change of BMD in physiologically normal rats as body homeostasis regulates the optimal levels of minerals in bone. Any values undergoing extreme changes may represent pathological stages rather than a beneficial effect. Another explanation is that a higher dose of bioactives may be required for efficacy; the dosage levels on a per‐kg body weight basis would have been reduced as food intake did not proportionally increase with increased body weight.

In addition, the bioactives in GSM may not be potent enough to prevent severe bone loss from OVX. Our results seem to support the benefit of omega‐3 rather than omega‐6 because the composition of the diets supplemented with GSM contained DHA and EPA, whereas these omega‐3 fatty acids were not present in the GSM‐free diets. Arachidonic acid is an omega‐6 fatty acid and was not present in any of the diet formulas. The total amount of DHA and EPA in ND + GSM was approximately 2 g/100 g. As the average daily feed intake of the rats was 20 g and the mean BW was 500 g, each rat would have received the combination of DHA and EPA at a dose of 800 mg/kg BW. This dose level is higher than the daily dosage recommended for humans^(^ ^41^ ^)^ and reached the reported therapeutic doses of GSM in animal studies,^(^ ^34^, ^36^, ^37^ ^)^ although these studies did not specifically assess bone health. Thus, it would be predicted that the daily intake in the current study would at least maintain the effective dose of DHA and EPA for anti‐inflammation in the rats. However, as individual lipids were not tested in this study, the bioactive factor(s) cannot be definitively identified. It is also noted that GSM diets contained more ash, and therefore it is possible that the beneficial effects of GSM included additive or synergistic effects between lipids, proteins and peptides, and/or minerals such as calcium.

Feeding rats with HFHS induced obesity, even though these animals consumed a lower volume of food because they had a higher overall energy intake due to the HFHS diet being energy‐dense. This resulted in a greater rate of weight gain per day. Weight gain and BW increased in accordance with both feed intake and energy intake in all groups except the sham rats on ND + GSM. Although those rats consumed less food than their paired diet group and even though the GSM diets provided slightly lower energy source (fat, protein, and carbohydrate) the rats' BW still increased significantly.

There was no significant change in the feed intake in all sham rats throughout the study. OVX rats, however, increased their food intake after the surgical procedure for a short period; within 2 months, their consumption rate reduced to these rats' baseline intake and matched the same level as sham animals. The modified food intake in OVX rats had significant effects on their weight gain and increased body weight. This main effect resulted in OVX rats being the most obese in the study; the brief period of increased food intake is likely due to the fact that ovarian hormones have direct effects on adipose tissues and indirect effect on regulation of feed intake and energy expenditure via hypothalamus.^(^ ^42^, ^43^ ^)^ Similar results were also found in other animal studies^(^ ^44^, ^45^ ^)^ and corresponding human studies in which postmenopausal women increased their food intake and weight a few years after the onset of menopause,^(^ ^46^ ^)^ alterations in metabolism that likely can be attributed to hormonal changes.

Fat mass was the source of BW gain in the older rats as can be found in the consecutive increase of percent change of body fat mass by DXA scans. Also, all main white fat pads harvested during necropsy (retroperitoneal, perigonadal, and inguinal fat pad) significantly increased from 6‐month‐old rats to 12‐month‐old rats. Inguinal fat, which is subcutaneous fat situated around groin and inguinal regions, was present as only a half of that perigonadal fat mass. In contrast to white fat, brown fat, a type of adipocyte responsible for utilizing energy expense through non‐shivering thermogenesis, has an inverse relationship with body mass index in human.^(^ ^47^ ^)^ There was a significant increase of interscapular brown fat due to feeding HFHS diet, but this effect was not present in OVX rats. This conflicting data may not be due to the sole effect of estrogen deficiency but rather combined effects of the hormone and HFHS diet resulting in severely impaired insulin sensitivity, which was revealed by significantly increased HbA1c in the OVX rats fed HFHS diet in our previous report^(^ ^48^ ^)^ and which influenced brown fat mass. Similar results of decreased brown fat mass were also revealed in obese male rats with insulin resistance.^(^ ^49^ ^)^ Moreover, insulin‐deprived animal models and insulin knockout rodents model demonstrated a decrease in brown fat mass.^(^ ^50^ ^)^

As expected, HFHS and OVX obviously showed cumulative effects on the expansion of these fat pads; therefore, the OVX rats on HFHS accumulated more fat tissue compared with the other groups. Interestingly, the inguinal fat pad, a type of subcutaneous fat composed of mixed functional (white and brown) adipocytes in OVX rats accelerated its expansion more pronounced than retroperitoneal and perigonadal fat pad, which are visceral fat composed of white adipocytes. This revealed that OVX influenced shifting the course of fat accumulation from visceral fat to inguinal fat. OVX did not only influence fat deposition but also significantly increased lean mass in a short period of time after surgery. It is possible that the observed increase in feed intake directly after ovariectomy supported an increase in body lean mass through increased protein intake. More importantly, the instant loss of estrogen due to ovariectomy would result in a higher androgen/estrogen ratio. The relative increase in androgens could enhance body lean mass and also influence the shift of fat distribution. This finding corroborates with Morita and colleagues^(^ ^51^ ^)^ reporting a short‐term increase of body lean mass in women after menopause and a shift of fat distribution from leg‐groin area to the upper part of body and abdominal cavity. There was an interaction effect of diet and surgical procedure that could possibly explain the different effects of OVX on the significant increase of fat mass only in ND groups but not HFHS groups at T3. Body fat tended to increase in sham rats but reduced in OVX rats when adding GSM into the diets, although these differences did not reach statistical significance when compared by one‐way ANOVA method within the same time. In contrast, body lean mass increased and reached the peak at the middle age of the animals, then declined at the older age.

Loss of BMD in OVX rats was observed consistently in the lumbar spine and femur scans. However, the whole‐body scan, which is less accurate for detecting bone loss in obese subjects with high body fat compared with the other scans, did not show reduced BMD at the end of the study in OVX rats. Thus using the whole‐body scan to interpret the relationship between body composition and BMD in the context of the obesity paradox may be inappropriate.

The other DXA scans revealed that the deposition of calcium in the femur, the long bone, takes precedence over the lumbar spine in rats as percent change of BMD in femur were higher compared with lumbar spine. Even though most bone sites showed significant bone loss in response to ovariectomy, the lumbar spine is the most sensitive bone site to detect the early bone loss in OVX rats. This finding seems to be different from the study of human trials indicating the total hip is the most reliable site for detecting osteoporosis or predicting hip fracture.^(^ ^52^ ^)^ This discrepancy might be due to the fact that our study scanned the whole femur, which is composed of more cortical bone rather than cancellous bone in the femoral head and pelvic part of the total hip region. However, the same study also recommended using spinal BMD for monitoring treatment. At around 6 months after OVX, estrogen deficiency could cause bone fragility in the lumbar spine as the BMD was lower than the baseline. The OVX rats fed HFHS lost BMD less than those fed ND in lumbar spine and right femur scans, indicating the benefit of obesity on reducing loss of BMD in OVX rats. It has been posited that the expansion of adipocytes in obesity may have an advantage on bone in postmenopausal women by being an alternative source of estrogen. Unfortunately, estrogen levels were not measured in this study and thus this cannot be confirmed, and a significant positive correlation between fat mass and BMD was not observed.

Bone health depends on the balance of bone metabolism, which is functioned by osteoblasts and osteoclasts. Many lines of evidence show that bone turnover rate (osteoclast function) increases after ovariectomy and results in bone loss.^(^ ^53^, ^54^ ^)^ However, our study found that the bone turnover markers TRAP and CTX‐I declined in the OVX rats. It is possible that adding HSHF diet in OVX rats could aggravate hyperglycemia and insulin resistance in rats as mentioned before, resulting in the reduction of bone metabolism. This finding corroborates with evidence in human with type 2 diabetes who have reduced bone remodeling markers such as procollagen type 1 N‐terminal propeptide, osteocalcin, alkaline phosphatase, and CTX‐I.^(^ ^55^, ^56^, ^57^ ^)^ In addition, hyperglycemia mice induced by high‐fat diet showed reduced biomechanical strength of trabecular and cortical bone with reduced expression of osteocalcin, as which the finding supported bone loss in hyperglycemia mice due to decreased bone formation (osteoblast function).^(^ ^58^ ^)^ Therefore, decreased bone metabolism is probably the underlying mechanism driving the reduction of BMD in our OVX rats. This finding reveals the fact that there may be different subtypes of osteoporosis in postmenopausal women based on the severity of metabolic conditions and, therefore, the specific therapeutic approach should be considered for those subtypes.

To explore some aspects of the complex relationship between obesity and bone and whether obesity has any benefit on BMD, relevant parameters of all rats at the endpoint data were pooled and correlation analysis was done. The data generally showed that it was not BW and fat mass but only lean mass that had a high correlation with BMD, although we observed significant correlations between fat mass and lean mass as well as between BW and fat mass and BW and lean mass. These findings demonstrate the complexity of defining “obesity” and might raise the question of whether BW needs to be adjusted for in correlation with BMD in obese populations as these subjects are likely to have increased fat mass proportionally higher than lean mass.

High levels of fat mass correlated with increased leptin production in the rats, which in turn was highly correlated with BW and fat mass, but leptin still failed to show a correlation with BMD. The data were stratified into groups and the correlations were reevaluated. Surprisingly, a highly positive correlation of BW and BMD in ND/sham rats was detected in all bone site scans and a similar result was found in the correlation with leptin. Therefore, in our model, increased BW and leptin correlated with an observed positive influence on BMD within the normal weight range. We hypothesize that there are likely to be causal relationships between some of these parameters, but this has not yet been demonstrated.

Many findings in this animal study correspond with the evidence in postmenopausal women. In women, bone growth during childhood and adolescence contributes to the achievement of peak bone mass, resulting in bone strength of later life.^(59^ ^)^ Attainment of peak bone mass depends on a variety of nutritional and endocrine factors.^(60^ ^)^ Many studies have reported the benefits of polyunsaturated fat (PUFA) consumption; however, omega‐3 and omega‐6 are debated in context of anti‐inflammation^(^ ^61^ ^)^ and bone health.^(62^ ^)^ The increase in BMD theoretically is related to increased calcium deposition in the bone matrix and this might be due to the capability of enhanced calcium absorption in duodenum by DHA and EPA.^(63^ ^)^

However, some discrepancies between rodents and humans were evident in this study or have been reported elsewhere. For instance, women have two main parts of subcutaneous fat, all located at the lower part of the body, which is gluteofemoral and abdominal fat depot. Gluteofemoral fat in women has been considered as a healthy fat associated with lower triglycerides^(^ ^64^ ^)^ and higher concentration of high‐density lipoprotein cholesterol,^(^ ^65^ ^)^ producing lower pro‐inflammatory cytokines than visceral fat,^(66^ ^)^ whereas abdominal fat has detrimental effects associated with insulin sensitivity similar to visceral fat.^(^ ^67^, ^68^ ^)^ A rat's subcutaneous fat, on the other hand is divided into cranial and caudal parts, which are interscapular fat and inguinal fat, respectively. Both fat pads have common type of beige adipocytes and different from white adipocytes of visceral fat. In postmenopausal women, preferential site of fat deposition is shifted from subcutaneous fat depot to visceral fat depot (intra‐abdominal fat) regardless of age,^(69^ ^)^ but this study showed that OVX rats increased inguinal fat mass more pronounced than visceral fat when compared with sham rats and, probably, total subcutaneous fat may be greater than visceral fat in OVX rats. Furthermore, women reach the peak of bone mass at the hip by approximately 20 years old^(^ ^9^ ^)^ followed by a decline, whereas rats are sexually mature by 2.5 months of age but bone growth still occurs until 10 months of age.^(^ ^70^ ^)^ BMD at all bone sites measured in the sham rats in this study increased over time; hence, the data may not extrapolate to human bone loss in advancing age. However, bone growth in this rat model provided an opportunity to investigate the effect of GSM on enhancement of calcium acquisition in bone.

In conclusion, our data have partially unraveled the ambiguity of the obesity paradox identified in humans and enabled inference with human bone health by establishing correlations between a number of parameters. First, increased body weight appears to enhance BMD to some extent as long as the weight gain is associated with an increase in lean mass. High fat mass in general was not beneficial to bone density in these rats, although it has been reported to have some advantages of increased bone mass in postmenopausal women. Moreover, consumption of GSM was correlated with increased acquisition of BMD in younger rats but has no effect on increased BMD in ovarian hormone deficiency.

## Disclosures

HST has a conflict of interest as she is employed by Sanford Limited, which is a mussel producer and exporter. All other authors state that they have no conflicts of interest.

5

### PEER REVIEW

The peer review history for this article is available at https://publons.com/publon/10.1002/jbm4.10571.

## Supporting information


**Appendix S1.** Supporting information

## Footnotes

1 ND = normal control diet; HFHS = high‐fat/high‐sugar diet; GSM = Greenshell mussel powder; ∑SFA = sum of saturated fatty acids; ∑MUFA = sum of monounsaturated fatty acids; ∑PUFA = sum of polyunsaturated fatty acids; ∑n‐3 PUFA = omega‐3 polyunsaturated fatty acids; ∑n‐6 PUFA = omega‐6 polyunsaturated fatty acids.

2 ND = normal control diet, HFHS = high‐fat/high‐sugar diet; OVX = ovariectomy; GSM = Greenshell mussel; BW = body weight. Time 0 = rat growing period on standard chow; Time 1 = experimental diets provided for 8 weeks; Time 2 = 8 weeks after the surgical procedure; Time 3 = termination of the study.
