# Supplementary material for: The Influence of Obesity, Ovariectomy, and Greenshell Mussel Supplementation on Bone Mineral Density in Rats
Source: JBMR Plus. 2021 Nov 14;6(1):e10571. doi: 10.1002/jbm4.10571 (PMC8771000; doi:10.1002/jbm4.10571)
Supplement: Supplementary file 1 — Appendix S1. Supporting information [file JBM4-6-e10571-s001.docx]

Supplemental Figure 6:

Correlation of body weight or leptin with bone mineral density in the individual group.

| **ND_Sham** | | |  |  |  |  |  |  |
| --- | --- | --- | --- | --- | --- | --- | --- | --- |
|  | | Body weight | Body fat mass | Body lean mass | Leptin (ng/ml) | whole body BMD | Right femur BMD | lumbar spine BMD |
| Body weight | Pearson Correlation | 1 | .857^**^ | .731^*^ | 0.549 | .902^**^ | .854^**^ | .831^**^ |
|  | Sig. (2-tailed) |  | 0.001 | 0.011 | 0.080 | 0.000 | 0.001 | 0.002 |
|  | N | 11 | 11 | 11 | 11 | 11 | 11 | 11 |
| Body fat mass | Pearson Correlation | .857^**^ | 1 | 0.300 | .776^**^ | .615^*^ | .722^*^ | .624^*^ |
|  | Sig. (2-tailed) | 0.001 |  | 0.369 | 0.005 | 0.044 | 0.012 | 0.040 |
|  | N | 11 | 11 | 11 | 11 | 11 | 11 | 11 |
| Body lean mass | Pearson Correlation | .731^*^ | 0.300 | 1 | 0.027 | .877^**^ | .644^*^ | .688^*^ |
|  | Sig. (2-tailed) | 0.011 | 0.369 |  | 0.937 | 0.000 | 0.033 | 0.019 |
|  | N | 11 | 11 | 11 | 11 | 11 | 11 | 11 |
| Leptin (ng/ml) | Pearson Correlation | 0.549 | .776^**^ | 0.027 | 1 | 0.328 | 0.330 | 0.221 |
|  | Sig. (2-tailed) | 0.080 | 0.005 | 0.937 |  | 0.325 | 0.321 | 0.513 |
|  | N | 11 | 11 | 11 | 11 | 11 | 11 | 11 |
| Whole body BMD | Pearson Correlation | .902^**^ | .615^*^ | .877^**^ | 0.328 | 1 | .855^**^ | .857^**^ |
|  | Sig. (2-tailed) | 0.000 | 0.044 | 0.000 | 0.325 |  | 0.001 | 0.001 |
|  | N | 11 | 11 | 11 | 11 | 11 | 11 | 11 |
| Right femur BMD | Pearson Correlation | .854^**^ | .722^*^ | .644^*^ | 0.330 | .855^**^ | 1 | .875^**^ |
|  | Sig. (2-tailed) | 0.001 | 0.012 | 0.033 | 0.321 | 0.001 |  | 0.000 |
|  | N | 11 | 11 | 11 | 11 | 11 | 11 | 11 |
| Lumbar spine_BMD | Pearson Correlation | .831^**^ | .624^*^ | .688^*^ | 0.221 | .857^**^ | .875^**^ | 1 |
|  | Sig. (2-tailed) | 0.002 | 0.040 | 0.019 | 0.513 | 0.001 | 0.000 |  |
|  | N | 11 | 11 | 11 | 11 | 11 | 11 | 11 |

Supplemental Figure 6:

(continue)

| ND_OVX |  |  |  |  |  |  |  |  |
| --- | --- | --- | --- | --- | --- | --- | --- | --- |
|  | | Body weight | Body fat mass | Body lean mass | Leptin (ng/ml) | whole body BMD | Right femur BMD | lumbar spine BMD |
| Body weight | Pearson Correlation | 1 | .966^**^ | .634^*^ | .878^**^ | 0.502 | .700^*^ | 0.081 |
|  | Sig. (2-tailed) |  | 0.000 | 0.027 | 0.000 | 0.096 | 0.011 | 0.803 |
|  | N | 12 | 12 | 12 | 12 | 12 | 12 | 12 |
| Body fat mass | Pearson Correlation | .966^**^ | 1 | 0.430 | .954^**^ | 0.553 | .696^*^ | 0.206 |
|  | Sig. (2-tailed) | 0.000 |  | 0.163 | 0.000 | 0.062 | 0.012 | 0.522 |
|  | N | 12 | 12 | 12 | 12 | 12 | 12 | 12 |
| Body lean mass | Pearson Correlation | .634^*^ | 0.430 | 1 | 0.212 | 0.306 | 0.555 | -0.150 |
|  | Sig. (2-tailed) | 0.027 | 0.163 |  | 0.508 | 0.334 | 0.061 | 0.641 |
|  | N | 12 | 12 | 12 | 12 | 12 | 12 | 12 |
| Leptin (ng/ml) | Pearson Correlation | .878^**^ | .954^**^ | 0.212 | 1 | 0.403 | 0.542 | 0.188 |
|  | Sig. (2-tailed) | 0.000 | 0.000 | 0.508 |  | 0.194 | 0.069 | 0.559 |
|  | N | 12 | 12 | 12 | 12 | 12 | 12 | 12 |
| Whole body BMD | Pearson Correlation | 0.502 | 0.553 | 0.306 | 0.403 | 1 | .758^**^ | .721^**^ |
|  | Sig. (2-tailed) | 0.096 | 0.062 | 0.334 | 0.194 |  | 0.004 | 0.008 |
|  | N | 12 | 12 | 12 | 12 | 12 | 12 | 12 |
| Right femur BMD | Pearson Correlation | .700^*^ | .696^*^ | 0.555 | 0.542 | .758^**^ | 1 | 0.333 |
|  | Sig. (2-tailed) | 0.011 | 0.012 | 0.061 | 0.069 | 0.004 |  | 0.291 |
|  | N | 12 | 12 | 12 | 12 | 12 | 12 | 12 |
| Lumbar spine_BMD | Pearson Correlation | 0.081 | 0.206 | -0.150 | 0.188 | .721^**^ | 0.333 | 1 |
|  | Sig. (2-tailed) | 0.803 | 0.522 | 0.641 | 0.559 | 0.008 | 0.291 |  |
|  | N | 12 | 12 | 12 | 12 | 12 | 12 | 12 |

Supplemental Figure 6:

(continue)

| ND+GSM_ Sham |  |  |  |  |  |  |  |  |
| --- | --- | --- | --- | --- | --- | --- | --- | --- |
|  | | Body weight | Body fat mass | Body lean mass | Leptin (ng/ml) | whole body BMD | Right femur BMD | lumbar spine BMD |
| Body weight | Pearson Correlation | 1 | .910^**^ | 0.571 | .937^**^ | 0.549 | 0.512 | -0.211 |
|  | Sig. (2-tailed) |  | 0.000 | 0.052 | 0.000 | 0.065 | 0.089 | 0.511 |
|  | N | 12 | 12 | 12 | 11 | 12 | 12 | 12 |
| Body fat mass | Pearson Correlation | .910^**^ | 1 | 0.198 | .863^**^ | 0.457 | 0.202 | -0.410 |
|  | Sig. (2-tailed) | 0.000 |  | 0.517 | 0.001 | 0.116 | 0.507 | 0.164 |
|  | N | 12 | 12 | 12 | 11 | 12 | 12 | 12 |
| Body lean mass | Pearson Correlation | 0.571 | 0.198 | 1 | .630^*^ | 0.337 | .621^*^ | 0.146 |
|  | Sig. (2-tailed) | 0.052 | 0.517 |  | 0.038 | 0.261 | 0.024 | 0.634 |
|  | N | 12 | 12 | 12 | 11 | 12 | 12 | 12 |
| Leptin (ng/ml) | Pearson Correlation | .937^**^ | .863^**^ | .630^*^ | 1 | 0.348 | 0.393 | -0.396 |
|  | Sig. (2-tailed) | 0.000 | 0.001 | 0.038 |  | 0.294 | 0.232 | 0.228 |
|  | N | 11 | 11 | 11 | 11 | 11 | 11 | 11 |
| Whole body BMD | Pearson Correlation | 0.549 | 0.457 | 0.337 | 0.348 | 1 | 0.372 | 0.445 |
|  | Sig. (2-tailed) | 0.065 | 0.116 | 0.261 | 0.294 |  | 0.210 | 0.128 |
|  | N | 12 | 12 | 12 | 11 | 12 | 12 | 12 |
| Right femur BMD | Pearson Correlation | 0.512 | 0.202 | .621^*^ | 0.393 | 0.372 | 1 | 0.510 |
|  | Sig. (2-tailed) | 0.089 | 0.507 | 0.024 | 0.232 | 0.210 |  | 0.075 |
|  | N | 12 | 12 | 12 | 11 | 12 | 12 | 12 |
| Lumbar spine_BMD | Pearson Correlation | -0.211 | -0.410 | 0.146 | -0.396 | 0.445 | 0.510 | 1 |
|  | Sig. (2-tailed) | 0.511 | 0.164 | 0.634 | 0.228 | 0.128 | 0.075 |  |
|  | N | 12 | 12 | 12 | 11 | 12 | 12 | 13 |

Supplemental Figure 6:

(continue)

| ND+GSM_OVX | | |  |  |  |  |  |  |
| --- | --- | --- | --- | --- | --- | --- | --- | --- |
|  | | Body weight | Body fat mass | Body lean mass | Leptin (ng/ml) | whole body BMD | Right femur BMD | lumbar spine BMD |
| Body weight | Pearson Correlation | 1 | .949^**^ | 0.270 | .840^**^ | .624^*^ | 0.252 | 0.229 |
|  | Sig. (2-tailed) |  | 0.000 | 0.395 | 0.001 | 0.030 | 0.429 | 0.475 |
|  | N | 12 | 12 | 12 | 12 | 12 | 12 | 12 |
| Body fat mass | Pearson Correlation | .949^**^ | 1 | -0.039 | .904^**^ | .581^*^ | 0.186 | 0.064 |
|  | Sig. (2-tailed) | 0.000 |  | 0.903 | 0.000 | 0.047 | 0.563 | 0.843 |
|  | N | 12 | 12 | 12 | 12 | 12 | 12 | 12 |
| Body lean mass | Pearson Correlation | 0.270 | -0.039 | 1 | -0.140 | 0.291 | 0.320 | .597^*^ |
|  | Sig. (2-tailed) | 0.395 | 0.903 |  | 0.664 | 0.359 | 0.311 | 0.040 |
|  | N | 12 | 12 | 12 | 12 | 12 | 12 | 12 |
| Leptin (ng/ml) | Pearson Correlation | .840^**^ | .904^**^ | -0.140 | 1 | 0.422 | 0.033 | 0.045 |
|  | Sig. (2-tailed) | 0.001 | 0.000 | 0.664 |  | 0.172 | 0.919 | 0.889 |
|  | N | 12 | 12 | 12 | 12 | 12 | 12 | 12 |
| Whole body BMD | Pearson Correlation | .624^*^ | .581^*^ | 0.291 | 0.422 | 1 | .700^*^ | 0.429 |
|  | Sig. (2-tailed) | 0.030 | 0.047 | 0.359 | 0.172 |  | 0.011 | 0.164 |
|  | N | 12 | 12 | 12 | 12 | 12 | 12 | 12 |
| Right femur BMD | Pearson Correlation | 0.252 | 0.186 | 0.320 | 0.033 | .700^*^ | 1 | .681^*^ |
|  | Sig. (2-tailed) | 0.429 | 0.563 | 0.311 | 0.919 | 0.011 |  | 0.015 |
|  | N | 12 | 12 | 12 | 12 | 12 | 12 | 12 |
| Lumbar spine_BMD | Pearson Correlation | 0.229 | 0.064 | .597^*^ | 0.045 | 0.429 | .681^*^ | 1 |
|  | Sig. (2-tailed) | 0.475 | 0.843 | 0.040 | 0.889 | 0.164 | 0.015 |  |
|  | N | 12 | 12 | 12 | 12 | 12 | 12 | 12 |

Supplemental Figure 6:

(continue)

| HFHS_ sham |  |  |  |  |  |  |  |  |
| --- | --- | --- | --- | --- | --- | --- | --- | --- |
|  | | Body weight | Body fat mass | Body lean mass | Leptin (ng/ml) | whole body BMD | Right femur BMD | lumbar spine BMD |
| Body weight | Pearson Correlation | 1 | .959^**^ | .629^*^ | .823^**^ | 0.516 | .732^**^ | 0.210 |
|  | Sig. (2-tailed) |  | 0.000 | 0.028 | 0.001 | 0.086 | 0.007 | 0.513 |
|  | N | 12 | 12 | 12 | 12 | 12 | 12 | 12 |
| Body fat mass | Pearson Correlation | .959^**^ | 1 | 0.393 | .881^**^ | 0.382 | .641^*^ | 0.237 |
|  | Sig. (2-tailed) | 0.000 |  | 0.206 | 0.000 | 0.221 | 0.025 | 0.459 |
|  | N | 12 | 12 | 12 | 12 | 12 | 12 | 12 |
| Body lean mass | Pearson Correlation | .629^*^ | 0.393 | 1 | 0.214 | .645^*^ | .657^*^ | 0.078 |
|  | Sig. (2-tailed) | 0.028 | 0.206 |  | 0.504 | 0.024 | 0.020 | 0.811 |
|  | N | 12 | 12 | 12 | 12 | 12 | 12 | 12 |
| Leptin (ng/ml) | Pearson Correlation | .823^**^ | .881^**^ | 0.214 | 1 | 0.197 | 0.374 | 0.269 |
|  | Sig. (2-tailed) | 0.001 | 0.000 | 0.504 |  | 0.539 | 0.231 | 0.398 |
|  | N | 12 | 12 | 12 | 12 | 12 | 12 | 12 |
| Whole body BMD | Pearson Correlation | 0.516 | 0.382 | .645^*^ | 0.197 | 1 | .716^**^ | 0.370 |
|  | Sig. (2-tailed) | 0.086 | 0.221 | 0.024 | 0.539 |  | 0.009 | 0.236 |
|  | N | 12 | 12 | 12 | 12 | 12 | 12 | 12 |
| Right femur BMD | Pearson Correlation | .732^**^ | .641^*^ | .657^*^ | 0.374 | .716^**^ | 1 | 0.353 |
|  | Sig. (2-tailed) | 0.007 | 0.025 | 0.020 | 0.231 | 0.009 |  | 0.261 |
|  | N | 12 | 12 | 12 | 12 | 12 | 12 | 12 |
| Lumbar spine_BMD | Pearson Correlation | 0.210 | 0.237 | 0.078 | 0.269 | 0.370 | 0.353 | 1 |
|  | Sig. (2-tailed) | 0.513 | 0.459 | 0.811 | 0.398 | 0.236 | 0.261 |  |
|  | N | 12 | 12 | 12 | 12 | 12 | 12 | 12 |

Supplemental Figure 6:

(continue)

| HFHS_ OVX |  |  |  |  |  |  |  |  |
| --- | --- | --- | --- | --- | --- | --- | --- | --- |
|  | | Body weight | Body fat mass | Body lean mass | Leptin (ng/ml) | whole body BMD | Right femur BMD | lumbar spine BMD |
| Body weight | Pearson Correlation | 1 | .990^**^ | .723^*^ | .787^**^ | 0.260 | .640^*^ | 0.420 |
|  | Sig. (2-tailed) |  | 0.000 | 0.012 | 0.007 | 0.439 | 0.034 | 0.198 |
|  | N | 11 | 11 | 11 | 10 | 11 | 11 | 11 |
| Body fat mass | Pearson Correlation | .990^**^ | 1 | .655^*^ | .805^**^ | 0.187 | 0.596 | 0.369 |
|  | Sig. (2-tailed) | 0.000 |  | 0.029 | 0.005 | 0.581 | 0.053 | 0.264 |
|  | N | 11 | 11 | 11 | 10 | 11 | 11 | 11 |
| Body lean mass | Pearson Correlation | .723^*^ | .655^*^ | 1 | 0.224 | 0.485 | .777^**^ | 0.372 |
|  | Sig. (2-tailed) | 0.012 | 0.029 |  | 0.534 | 0.130 | 0.005 | 0.260 |
|  | N | 11 | 11 | 11 | 10 | 11 | 11 | 11 |
| Leptin (ng/ml) | Pearson Correlation | .787^**^ | .805^**^ | 0.224 | 1 | 0.102 | 0.419 | 0.532 |
|  | Sig. (2-tailed) | 0.007 | 0.005 | 0.534 |  | 0.778 | 0.229 | 0.114 |
|  | N | 10 | 10 | 10 | 10 | 10 | 10 | 10 |
| Whole body BMD | Pearson Correlation | 0.260 | 0.187 | 0.485 | 0.102 | 1 | .654^*^ | .609^*^ |
|  | Sig. (2-tailed) | 0.439 | 0.581 | 0.130 | 0.778 |  | 0.029 | 0.047 |
|  | N | 11 | 11 | 11 | 10 | 11 | 11 | 11 |
| Right femur BMD | Pearson Correlation | .640^*^ | 0.596 | .777^**^ | 0.419 | .654^*^ | 1 | .785^**^ |
|  | Sig. (2-tailed) | 0.034 | 0.053 | 0.005 | 0.229 | 0.029 |  | 0.004 |
|  | N | 11 | 11 | 11 | 10 | 11 | 11 | 11 |
| Lumbar spine_BMD | Pearson Correlation | 0.420 | 0.369 | 0.372 | 0.532 | .609^*^ | .785^**^ | 1 |
|  | Sig. (2-tailed) | 0.198 | 0.264 | 0.260 | 0.114 | 0.047 | 0.004 |  |
|  | N | 11 | 11 | 11 | 10 | 11 | 11 | 11 |

Supplemental Figure 6:

(continue)

| HFHS+ GSM_ sham |  |  |  |  |  |  |  |  |
| --- | --- | --- | --- | --- | --- | --- | --- | --- |
|  | | Body weight | Body fat mass | Body lean mass | Leptin (ng/ml) | whole body BMD | Right femur BMD | lumbar spine BMD |
| Body weight | Pearson Correlation | 1 | .911^**^ | 0.540 | .771^**^ | 0.440 | .873^**^ | 0.054 |
|  | Sig. (2-tailed) |  | 0.000 | 0.057 | 0.002 | 0.132 | 0.000 | 0.861 |
|  | N | 12 | 12 | 12 | 12 | 12 | 12 | 12 |
| Body fat mass | Pearson Correlation | .911^**^ | 1 | 0.155 | .922^**^ | 0.143 | .724^**^ | -0.183 |
|  | Sig. (2-tailed) | 0.000 |  | 0.612 | 0.000 | 0.642 | 0.005 | 0.549 |
|  | N | 12 | 12 | 12 | 12 | 12 | 12 | 12 |
| Body lean mass | Pearson Correlation | 0.540 | 0.155 | 1 | -0.052 | .765^**^ | .635^*^ | .563^*^ |
|  | Sig. (2-tailed) | 0.057 | 0.612 |  | 0.866 | 0.002 | 0.020 | 0.045 |
|  | N | 12 | 12 | 12 | 12 | 12 | 12 | 12 |
| Leptin (ng/ml) | Pearson Correlation | .771^**^ | .922^**^ | -0.052 | 1 | -0.039 | .603^*^ | -0.308 |
|  | Sig. (2-tailed) | 0.002 | 0.000 | 0.866 |  | 0.898 | 0.029 | 0.307 |
|  | N | 12 | 12 | 12 | 12 | 12 | 12 | 12 |
| Whole body BMD | Pearson Correlation | 0.440 | 0.143 | .765^**^ | -0.039 | 1 | .695^**^ | .818^**^ |
|  | Sig. (2-tailed) | 0.132 | 0.642 | 0.002 | 0.898 |  | 0.008 | 0.001 |
|  | N | 12 | 12 | 12 | 12 | 12 | 12 | 12 |
| Right femur BMD | Pearson Correlation | .873^**^ | .724^**^ | .635^*^ | .603^*^ | .695^**^ | 1 | 0.440 |
|  | Sig. (2-tailed) | 0.000 | 0.005 | 0.020 | 0.029 | 0.008 |  | 0.132 |
|  | N | 12 | 12 | 12 | 12 | 12 | 12 | 12 |
| Lumbar spine_BMD | Pearson Correlation | 0.054 | -0.183 | .563^*^ | -0.308 | .818^**^ | 0.440 | 1 |
|  | Sig. (2-tailed) | 0.861 | 0.549 | 0.045 | 0.307 | 0.001 | 0.132 |  |
|  | N | 12 | 12 | 12 | 12 | 12 | 12 | 12 |

Supplemental Figure 6:

(continue)

| HFHS+ GSM_ OVX |  |  |  |  |  |  |  |  |
| --- | --- | --- | --- | --- | --- | --- | --- | --- |
|  | | Body weight | Body fat mass | Body lean mass | Leptin (ng/ml) | whole body BMD | Right femur BMD | lumbar spine BMD |
| Body weight | Pearson Correlation | 1 | .962^**^ | 0.345 | .734^**^ | -0.111 | 0.470 | -0.129 |
|  | Sig. (2-tailed) |  | 0.000 | 0.299 | 0.007 | 0.744 | 0.145 | 0.706 |
|  | N | 12 | 11 | 11 | 12 | 11 | 11 | 11 |
| Body fat mass | Pearson Correlation | .962^**^ | 1 | 0.094 | .879^**^ | -0.205 | 0.316 | -0.298 |
|  | Sig. (2-tailed) | 0.000 |  | 0.783 | 0.000 | 0.545 | 0.344 | 0.373 |
|  | N | 11 | 11 | 11 | 11 | 11 | 11 | 11 |
| Body lean mass | Pearson Correlation | 0.345 | 0.094 | 1 | 0.146 | 0.386 | .759^**^ | .684^*^ |
|  | Sig. (2-tailed) | 0.299 | 0.783 |  | 0.668 | 0.242 | 0.007 | 0.020 |
|  | N | 11 | 11 | 11 | 11 | 11 | 11 | 11 |
| Leptin (ng/ml) | Pearson Correlation | .734^**^ | .879^**^ | 0.146 | 1 | -0.214 | 0.222 | -0.271 |
|  | Sig. (2-tailed) | 0.007 | 0.000 | 0.668 |  | 0.528 | 0.512 | 0.420 |
|  | N | 12 | 11 | 11 | 12 | 11 | 11 | 11 |
| Whole body BMD | Pearson Correlation | -0.111 | -0.205 | 0.386 | -0.214 | 1 | 0.556 | .621^*^ |
|  | Sig. (2-tailed) | 0.744 | 0.545 | 0.242 | 0.528 |  | 0.076 | 0.042 |
|  | N | 11 | 11 | 11 | 11 | 11 | 11 | 11 |
| Right femur BMD | Pearson Correlation | 0.470 | 0.316 | .759^**^ | 0.222 | 0.556 | 1 | .673^*^ |
|  | Sig. (2-tailed) | 0.145 | 0.344 | 0.007 | 0.512 | 0.076 |  | 0.023 |
|  | N | 11 | 11 | 11 | 11 | 11 | 11 | 11 |
| Lumbar spine_BMD | Pearson Correlation | -0.129 | -0.298 | .684^*^ | -0.271 | .621^*^ | .673^*^ | 1 |
|  | Sig. (2-tailed) | 0.706 | 0.373 | 0.020 | 0.420 | 0.042 | 0.023 |  |
|  | N | 11 | 11 | 11 | 11 | 11 | 11 | 11 |
